# Progress in Research and Application of Metal–Organic Gels: A Review

**DOI:** 10.3390/nano13071178

**Published:** 2023-03-25

**Authors:** Gen Liu, Siwen Li, Chunyan Shi, Mingxin Huo, Yingzi Lin

**Affiliations:** 1 School of Environment, Northeast Normal University, Changchun 130117, China; 2 Engineering Lab for Water Pollution Control and Resources Recovery, School of Environment, Northeast Normal University, Changchun 130117, China; 3 Faculty of Environmental Engineering, The University of Kitakyushu, Kitakyushu 808-0135, Japan; 4 School of Municipal & Environmental Engineering, Jilin Jianzhu University, Changchun 130118, China; 5 Key Laboratory of Songliao Aquatic Environment, Ministry of Education, Jilin Jianzhu University, Changchun 130118, China

**Keywords:** MOGs, citespace, adsorption, catalysis, application

## Abstract

In recent years, metal–organic gels (MOGs) have attracted much attention due to their hierarchical porous structure, large specific surface area, and good surface modifiability. Compared with MOFs, the synthesis conditions of MOGs are gentler and more stable. At present, MOGs are widely used in the fields of catalysis, adsorption, energy storage, electrochromic devices, sensing, analysis, and detection. In this paper, literature metrology and knowledge graph visualization analysis are adopted to analyze and summarize the literature data in the field of MOGs. The visualization maps of the temporal distribution, spatial distribution, authors and institutions’ distribution, influence of highly cited literature and journals, keyword clustering, and research trends are helpful to clearly grasp the content and development trend of MOG materials research, point out the future research direction for scholars, and promote the practical application of MOGs. At the same time, the paper reviews the research and application progress of MOGs in recent years by combining keyword clustering, time lines, and emergence maps, and looks forward to their challenges, future development trend, and application prospects.

## 1. Introduction

With the continuous development of global industrialization, many environment-related problems have arisen, which pose a great threat to human health and the ecological environment. In order to solve these environmental problems, a variety of new materials have been developed one after another. Many environment-related problems are related to nanoporous materials, which have great potential value in pollutant removal, gas storage, and catalysis due to their complex porous structure and large specific surface area [1,2]. In recent years, a new kind of porous material, metal–organic gels (MOGs), has appeared in the intersection of inorganic chemistry and coordination chemistry. MOGs are a kind of metal–organic polymer formed by metal ions and organic ligands through coordination bonding, van der Waals forces, hydrogen bonding,π–π accumulation or other intermolecular forces [3,4]. They have the advantages of large surface area, high porosity, and abundant surface functional groups. Different from MOFs, MOGs are easier to synthesize, green, and have mild reaction conditions. There are two main stages in the formation of MOGs. The first stage is the aggregation of metal ions and ligands to form MOF clusters, which are then polymerized from prepolymers to form nuclei. In the second stage, due to the agitation of coordination balance, amorphous branches of MOF particles occur, leading to crosslinking and contributing to gelation [5]. Metal–organic gels show a semi-solid state because metal ions and ligands form a specific three-dimensional network structure in the process of interaction, which hinders the flow of solvents so that a large number of solvent molecules are wrapped in the internal structure of the system, thus transforming the initial liquid state into non-flowing and stable semi-solid-state substances. Ligand-based gels can confer other physicochemical properties of metal ions, such as magnetism, color, rheology, adsorption, emission, photophysical properties, catalytic activity, and oxidation-reduction behavior, and ligands can also provide corresponding functional groups [6,7,8,9]. Both metal–organic gels and metal–organic frameworks are materials composed of metal ions and organic ligands. They have hierarchical porous structure, high specific surface area, and surface modifiability, but they are different in structure, properties, and applications. First of all, metal–organic gels do not have a crystal structure but are composed of a gel system with a three-dimensional network structure, in which metal ions and organic ligands are connected together through coordination bonds, forming a sponge-like structure. The metal–organic framework has a crystal structure, which is a two-dimensional or three-dimensional structure composed of metal ions and organic ligands, in which metal ions and organic ligands are connected by coordination bonds to form a grid-like structure. Secondly, metal–organic gels have high porosity and surface area, which can adsorb and store molecules such as gases, liquids, and ions. Metal–organic frameworks also have high porosity and surface area, but their structure is more stable and can be used in extreme conditions such as high temperature and pressure. Finally, metal–organic gels are mainly used in gas separation, catalytic reaction, drug delivery, and other fields. Metal–organic frameworks are mainly used in gas storage, separation, sensors, catalytic reactions, and other fields. In general, metal–organic gels and metal–organic frameworks are new materials with wide application prospects. Their different structures and properties also determine their applications in different fields.

**Figure 1:**
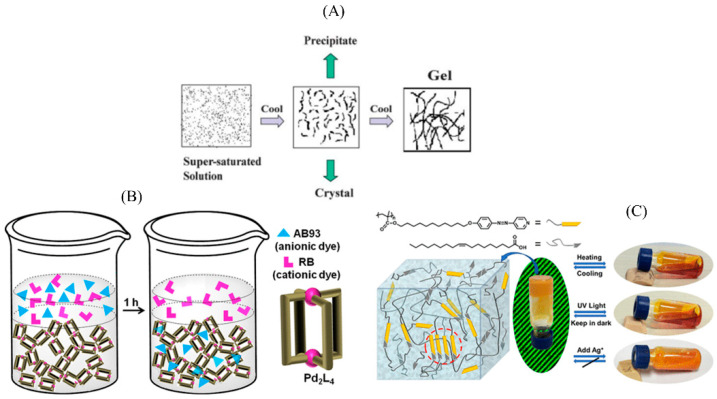
Schematic diagram of organomemetal gel aggregation process [18]. (**A**) Pd_2_L_4_ gel factor formation process [16]; (**B**) PM_11_A_z_P_y_ gel factor formation process [17] (**C**).

At the early stage of research, some scholars called MOGs as inorganic–organic hybrid semi-solid substances or supramolecular gels. Therefore, combining the views of Flory [10], Steed [11],Tam [12] et al., MOGs were divided into two categories according to the composition, structure, and synthesis of metal–organic gels. The first category is gel factor type metal–organic gels, which consist of metal ions and organic ligands interacting in a solvent to form gel factors (complexes), which then solidifies the solvent to form a gel under non-covalent bonds (hydrogen bonds, π–π interactions, van der Waals forces). Metal ions are part of the gel factor, and the introduction of metal ions is only to improve the physical and chemical properties of the gel and enhance its strength; the coordination is not the main force in the process of gel formation. Terech et al. [13] first found that zinc (II) and porphyrins formed rod-like aggregations in cyclohexane solution through electron microscopy, small-angle neutron scattering, and small-angle X-ray scattering techniques, namely, the gel factor ZnP_3_. Shinkai et al. [14] reported that a new metal–organic gel 1-PrCN gel was formed by solvent gelation of Cu(Ⅰ) and gel factor Cu(Ⅰ)•12 formed by 2,2′-bipyridine derivatives with two cholesterol groups. Lam et al. [15] synthesized a series of Re(Ⅰ) complexes, all of which can form a gel in dimethyl sulfoxide (DMSO). During the formation of the gel, coordination does not play a major driving role. As shown in Figure 1, Dillp et al. [16] constructed a Pd_2_L_4_ gel factor through the complexation of Pd(II) with N, N′-bis (3-pyridylmethyl)-naphthalenediimide (L), which was dissolved in dimethyl sulfone or acetonitrile-water (1:1) and then left at room temperature. It showed further self-assembly, forming a gel phase. Synergies between metal ions, ligands, counter-ions, solvents, and concentrations were observed to play a crucial role in the formation of metal gels. Notable features of gels include thixotropy and reversible chemical stimulus reaction behavior. Porous gels show the ability to absorb pyrene as a guest and selectively remove anionic dyes from aqueous solutions. Yu et al. [17] reported self-assembly of SLCP organogels using azopyridine-containing polymers (PM_11_A_z_P_y_) and oleic acid (OA) as gel factors. In particular, oleic acid is not only an important component in the construction of mesocrytic gels but also an interstitial solvent in physically crosslinked three-dimensional (3D) network gaps.

The other kind of gel was the coordination polymer metal–organic gel, which is formed through the coordination between metal ions and ligands. Its essence is a coordination polymer without gel factor, and the main driving force of gel formation is the coordination between metal and ligands. Therefore, the coordination polymer gel has higher metal content and better stability [19,20]. The formation process of coordination polymer metal–organic gels is generally as follows: a certain proportion of metal ions and organic ligands in the solvent is weighed and heated to the isotropic dissolution state; at this time the molecular aggregation will show the following three states: one is the formation of highly ordered crystals, namely, metal–organic framework (MOFs); second, molecular aggregation precipitates form amorphous complexes; the third is an in-between state, forming metal–organic gels (MOGs) between solid and liquid [21]. Naota et al. [22] constructed metal–organic gels by using the coordination between Pd(II) and a heterocycle molecule containing N and O, and proposed that the coordination between Pd(II) and ligand occurred at room temperature, but gel could not be formed due to the curvature of ligand molecules. After ultrasound, the ligand molecules changed from curved to flat, forming interlocking structures under the action of molecular accumulation and then forming gels. Su et al. [23] used terephthalic acid (TPA) to coordinate with A1^3+^ to form a metal–organic gel, and found that A1^3+^ was pre-assembled with TPA in solution to form a crystal nucleus. After that, if the coordination continues and the crystal continues to grow, MOF will be obtained; if the coordination is interrupted at this time, the mismatch will occur and the metal–organic gel will be obtained. Wei et al. [24] proposed the following reversible solution–gel transformation mechanism with the synthesis of metal–organic gels based on Al(NO_3_)_3_•9H_2_O and DCBTF6: Ligands DCBTF6 and Al^3+^ ions form nanoscale clusters or particles through coordination interactions, assisted by the formation of other supramolecular interactions including hydrogen bonds. This coordination equilibrium is sensitive to temperature changes. Appropriate heating promotes the aggregation of metal–organic nanoparticles and enhances stronger coordination interactions to form gels. Lowering the temperature reverses this process, allowing other supramolecular interactions to dominate, thus dissociating the gel skeleton. Botian et al. [25] synthesized a new type of heat-resistant metal–organic gel based on Fe (NO_3_) _3_ and bis (3-pyridyl) terephthalate. Based on gold ligand coordination, gelling showed high selectivity for Fe (III). Shinkai et al. reported that the metal coordination polymer gel prepared by 1,2,4,5-tetra (2H-tetrazole-5-yl)-benzene (TTB) and Co^2+^ can be used as a chemical sensor for chlorine-containing molecules [26].

In recent years, bibliometrics has become an important means of research progress in various academic fields [27,28], that is, quantitative analysis of scientific papers in a certain field with statistical and mathematical methods, combined with visualization technology, intuitive display of research development history, research status, research hotspots and development trend of the topic, and prediction of emerging research trends. Used to explore the research hotspot and direction [29,30]. This approach allows us to explore and analyze large amounts of scientific data through performance indicators to identify the major contributions of authors, institutions, and journals in productivity, citations, and bibliographic coupling [31]. At present, the number of MOGs published in water treatment, catalysis, medical treatment, fluorescence detection, and other fields is increasing year by year, but the lack of systematic analysis makes it difficult to grasp the research focus of metal–organic gels. In recent years, researchers have introduced metal ions, metal–organic compounds, and metal nanoparticles into gel networks, and induced the formation of metal–organic gels (MOGs) by the non-covalent interaction between metal ions and organic ligands. Combining the unique characteristics of metal components (oxidization and reduction reaction, optical, electric, and magnetic) with the characteristics of organic gels, MOGs have the advantages of large specific surface area, tunable structure, rich metal sites, and good stability, and are widely used in the fields of sensing, adsorption, catalysis, sewage treatment, and dye adsorption. Therefore, in order to further understand the research hotspots and development trend of MOGs in the field of water treatment, the literature metrology method was adopted to search the papers related to metal–organic gels in the core database of Web of Science (WOS). With the help of visualization software citespace, the intuitive map was drawn to deepen the research status of metal–organic gels, reveal the research hotspots and development trend, and provide some reference for the exploration of the frontier issues in the field of metal–organic gels. The main research contents are as follows: (1) MOGs publishing trends, sources, journals, disciplinary distribution, as well as leading authors, countries, and research institutions; (2) collaboration between the lead author and the institution in the field of MOGs research; (3) summary of existing hot issues, exploration of research prospects and trends based on keyword cluster analysis and time graph and keyword outburst graph analysis. It is of great significance to comprehensively understand and promote the research and application of MOGs.

## 2. Methods

### 2.1. Data Acquisition

Web of Science (WOS) is an important database platform for obtaining global academic information [32]. The data in this paper are from the core collection of Web of Science (WOS). The edition is set as “SCI-EXPANDED”, and the time span is “ALL YEARS”. It contains more records than other academic databases, but mainly by generating more complete original data for bibliometric analysis software [33]. The search formula is: Subject search = (Metal Organic Gel*) OR (Metal Organic Gels* OR Metal-Organic Gel*) OR (Metal-Organic Gels*) OR (Supramolecular organic gels*)OR (Supramolecular organic gels*) (* indicates 0 or more characters); The language was selected as “English” and the literature type was selected as “article” and “review”. A total of 2209 records were retrieved. After excluding articles with irrelevant topics, not obvious co-citation relationship and lack of innovation, only 537 records met the requirements. The research in the field of metal–organic gels is still in the early stage of development, and the current research results are indeed based on a relatively small database, which is also one of our limitations. However, in the study, we adopted a strict bibliometric analysis method to ensure the reliability of the conclusions. They were downloaded in plain text format for “Full record and reference” and the data were analyzed by citespace.

### 2.2. Methods

The literature measurement software citespace developed by Professor Chaomei Chen from the School of Information Science and Technology of Drexel University was used. Based on the above Web of Science source data and bibliometric and visual analysis of 537 MOGs studies, key information was extracted to build a co-occurrence network. In citespace, annual publication amount, national publication amount, citation frequency and time line of author/institution, citation frequency of literature co-citation, relationship between subject and topic, keyword co-occurrence, clustering and time line, keyword time graph, and keyword prominence are drawn. In all the graphs, the size of the node represents the frequency of occurrence; the larger the node, the higher the frequency; the color of the node represents the chronological order; from the bottom (dark purple) to the top (light yellow) represents the past to the present, and each color in the circle corresponds to a color band. The area of the circle is proportional to the frequency of the target (such as country, institution, author, citation) of the year represented by the corresponding color. Intermediary centrality refers to the ratio of the shortest path connecting two nodes through a certain point in the network to the total number of shortest path lines between the two nodes. If the intermediary centrality is greater than 0.1, the node is the key node of the network. In addition, all the relevant parameters in the mapping process can be obtained in the upper left of the corresponding figure.

## 3. Results and Discussion

### 3.1. Literature Quantity Analysis

**Figure 2:**
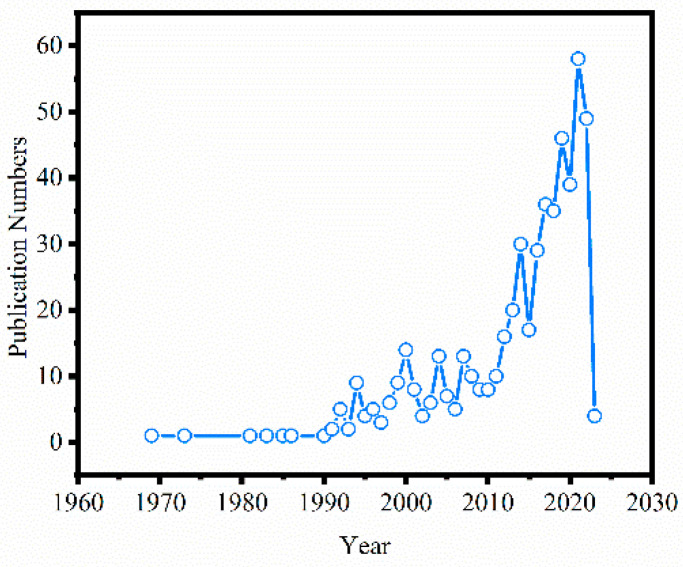
Change of annual number of papers in the field of metal–organic gels based on Web of Science.

The number of publications in a field is a good proxy measure of knowledge generation, so we statistically analyzed the number of articles in recent years to investigate trends in the field of metal–organic gels [34]. Figure 2 shows the development process of 537 papers on metal–organic gels published from 1960 to 2023. According to the growth trend of the number of papers published, we can see the degree of attention people pay to metal–organic gels. In order to better show the degree of attention people pay to metal–organic gels, this paper divides the research time into three periods: the initial stage of research (1960–1990), the initial development stage of research (1991–2010), and the rapid growth stage of research (2011–2023). In the initial stage of research, only one article was published after several years. In this period, there were only a few reports on the synthesis and properties of MOGs. At the initial stage of development, more than two articles on metal–organic gels were published every year, mainly focusing on the synthesis and simple application of MOGs. At the stage of rapid growth of research, the number of papers published has increased to two digits per year. The research direction pays more attention to the innovation of the synthesis methods of MOGs, and the powder sizing, application range, and removal mechanism are more extensive. The increasing number of publications year by year means that the field of MOGs is in a stage of rapid development and has attracted more and more attention from international scholars.

### 3.2. Analysis of Publication Countries 

**Figure 3:**
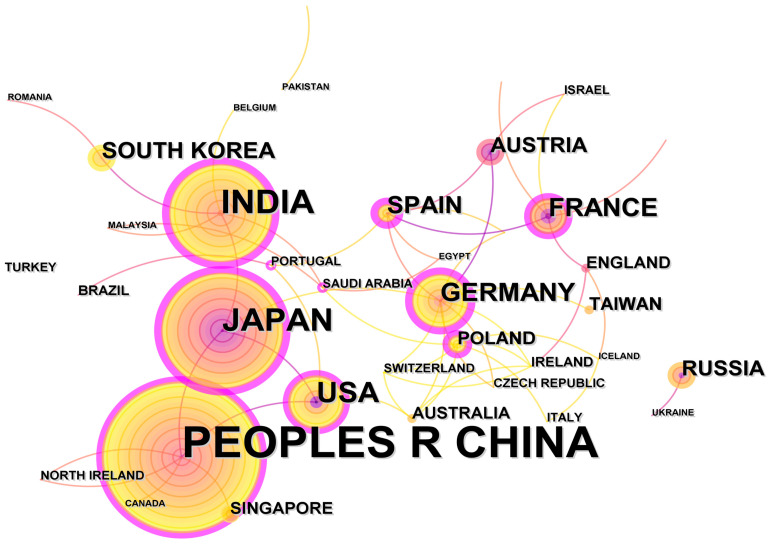
Network of countries affiliated to MOGs.

Figure 3 is the result of running the software on the node “country”, and Table 1 is based on the statistics of the top ten countries in terms of the number of MOGs-related publications. The number of countries appearing is represented by their frequency. As shown in Figure 3, the People’s Republic of China is the country with the largest number of publications, followed by Japan, India, the United States, France, Germany, Spain, and other countries. The first countries to do so were the United States in 1993 and India in 1994, then France in 1995, China in 1998, Spain and Russia in 1999, and Japan in 2001.The rest of the countries have published MOGs since 2000. The position of a country in this field is indicated by the degree of centrality. The more countries are connected, the more centered the country is, the richer the country’s research will be, and the more important the country’s position will be. China’s centrality is 0.31, which indicates that China has made significant contributions in the field of MOGs and has close cooperation with other countries. It can be seen from Figure 3 that China is directly connected with the United States, Japan, Singapore, Canada, and other countries, and India, Germany, France, and Spain are indirectly connected as well, which shows that China is inclined to communicate and cooperate with other countries, makes a great contribution to the cooperation between countries, and its development status is in a leading position

### 3.3. Cooperation between Scientific Research Authors and Institutions

**Figure 4:**
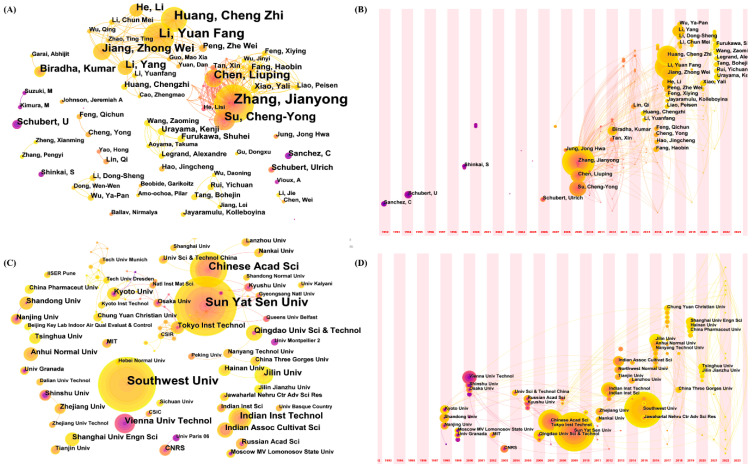
Network diagram of affiliated authors in MOGs. (**A**) Time diagram of affiliated authors in MOGs (**B**) Network diagram of affiliated organizations in MOGs. (**C**) Time diagram of affiliated organizations in MOGs (**D**).

The number of articles published by the author and the research institution can reflect the research status of the corresponding author and institution in the field, and also represent the researchers and institutions that have made great academic contributions to the field during this period. At the same time, it can reflect the distribution of academic resources in the field. To this end, this paper makes a detailed analysis of the data information of the author and the institution. Figure 4A,C take “author” and “institution” as analysis fields, respectively. In the figure, each node corresponds to the author or institution, and the lines represent the cooperative relationship between the author/institution. Table 2 shows the top 10 authors in the field of MOGs, among which Zhang Jianyong, Li YuanFang and Huang Cheng Zhi are the authors with the most published articles, reaching more than 20. At the same time, the authors cooperated closely. Figure 4A shows that Zhang Jianyong and Su Cheng-yon, Li YuanFang and Huang Cheng Zhi were connected more, and a certain scale of academic community was formed. Zhang Jianyong and Su Cheng-Yong are both from Sun Yat-sen University, and Li YuanFang and Huang ChengZhi are from Southwest University. Core authors are those who have published more papers and have greater influence in the research field. According to Plath’s law, the number of core authors’ published papers can be calculated according to formula (1):

(1) $$N=0.749\sqrt{\eta_{\max}}$$

In the formula, N is the number of published papers by core authors, which is the highest number of published papers. Therefore, there are 36 core authors with more than four published papers, and the number of published papers is 279, accounting for nearly 50%. This indicates that although the core authors in the field of MOGs have a high productivity, the core authors have not yet formed.

The analysis of the issuing institution (Table 3) shows that Sun Yat-sen University, Southwest University, and Chinese Acad Sci are in the most prominent position in the field of MOGs, which is consistent with the author’s conclusion. Indian Inst Technol, Vienna Univ Technol, Jilin University, Qingdao Univ. Sci & Technol, The Indian Assoc Cultivat Sci, and other institutions also have significant contributions to research in the MOGs field. Figure 4C shows that at present, there is less cooperation among institutions, mainly concentrated in Kyoto university, Osaka University, and Kyushu University, which is not conducive to the rapid development of this field, so it is necessary to strengthen cooperation among institutions. In terms of the number of articles published by institutions, the total number of articles published by the top four institutions is more than 10, among which the number of articles published by Sun Yat-sen University, Southwest University, and Chinese Acad Sci is more than other institutions.

Figure 4B,D are about the time zone chart with “author” and “institution”, respectively. The time zone chart is used to study the changes of “author” and “institution” over time. Each circle in the figure represents an author/institution, and the keyword is the year when it first appears in the analyzed data set. Although the author/field will still appear later, it will only increase the frequency by one in the newly appeared position. Figure 4B shows that in the existing data set, Sanchez C is the first author to publish relevant literature, and Su Cheng-Yon, Chen LiuPing, and Zhang Jianyong appeared for the first time in 2009 and belong to the core group of authors. After that, Huang Chenzhi, Li YuanFang, Jiang Zhong Wei and others published several articles in the field of MOGs. Figure 4D shows Kyoto University, Shandong University, Nanjing University, and Sun Yat-sen University, which published an article in the field of MOGs for the first time in the data set. Southwest University, Chinese Acad Sci, and other institutions with high frequency published in 2009, 2007, and 2015, respectively.

### 3.4. Co-Citation Analysis

**Figure 5:**
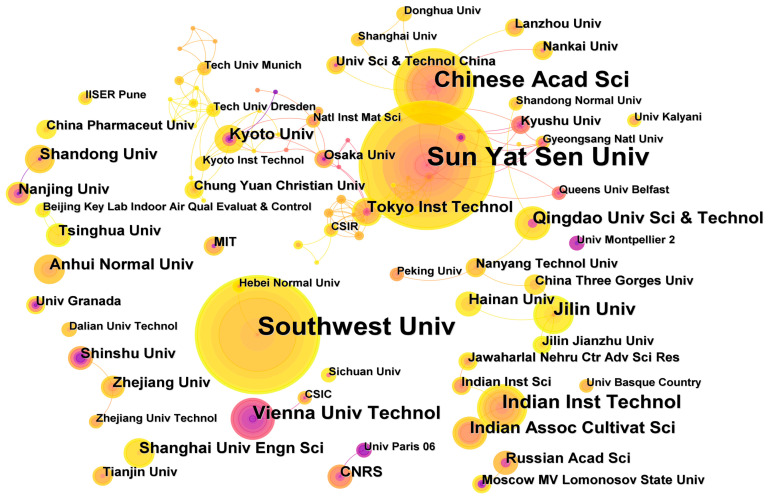
Co-citation analysis diagram of MOGs.

Co-citation analysis can help readers quickly identify which literatures are read and cited in the research field [35,36]. When two or more papers are published, they are cited by one or more papers in later relevant studies, which constitutes the co-cited literature relationship. The more co-citation times, the greater the similarity between the two literatures and the greater the correlation strength. As shown in Table 4 and Figure 5, the most cited paper is Piepenbrock’s 2010 Chemical Reviews article “Metal- and ion-Binding Supramolecular Gels [11].” Citation frequency was 72 times, and this paper mainly reviewed that adding metals and anions to gel factors can further adjust the properties of the material, including its optical, magnetic, self-assembly, morphology, and rheological behavior, so as to be applied to different fields. Secondly, Cheng-Yong Su published “A synthetic route to ultralight hierarchically micro/mesoporous Al(III)-carboxylate [37]“ in NAT COMMUN in 2013. In this paper, they mainly studied the step-to-step gelation based on MOF nanoparticles, and proved that the production of various hierarchical porous Al (III)-MOA has the characteristics of high surface area, low density, and adjustable porosity. The hierarchical micro/mesoporous Al organomemetal aerogel was thoroughly evaluated by N_2_ adsorption. The good accessibility of micro/mesopore was verified by steam/dye absorption, and their potential as effective fiber coating absorbers was tested in solid phase microextraction analysis; Cheng-Yong Su also published in COORDIN CHEM REV that same year: “Metal-organic gels: From discrete metallogelators to coordination polymers” [38]; the design and properties of various types of metal gels were introduced. Design strategies were developed based on organic gels, including metal incorporation into low molecular weight organic gels (LMWG). Coordination polymers were assisted by auxiliary components, including lipophilic and hydrogen bonding groups, as gels. In addition, a new class of metal–organic gels without auxiliary parts was developed. These coordination-oriented (induced) gels show novel properties, for example in adsorption and catalysis, and as templates for porous materials. Table 1 lists the cited authors, cited times, and journals of the top 10 highly cited literatures, which have made important contributions to the innovation and development of MOGs.

### 3.5. The Relationship between Disciplines and Journals

The double-graph overlay of journals shows the distribution of papers on the subject relative to each subject, with each dot representing a journal. On the left is the application map, representing the research status; on the right is the cited map, representing the research basis; the curve is the citation line, reflecting the source and place of the literature, and the width of the connection curve reflects the similarity degree of the subject. The internal numbers in the ellipse represent the number of publications in each discipline [46].

**Figure 6:**
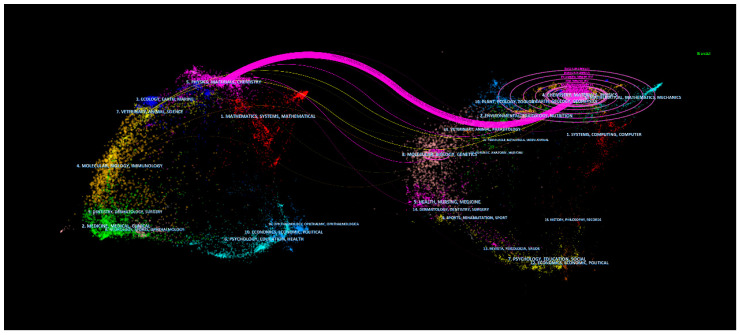
Double-map overlay for MOGs research.

As shown in Part A on the left of Figure 6, the research status of metal–organic gels focuses on the research literature of physics, materials, chemistry, etc., and the right half takes the discipline of the cited literature as the research basis of metal–organic gels. Citation fields focus more on physics, materials, chemistry, mathematics, mechanics, ecology, and environment [47]. It is worth noting that in the citation field on the left, physics, materials, and chemistry have an outward citation path, indicating that this discipline is the most dominant citation discipline. The pink line puts the study of metal–organic gels across a wide range of disciplines, with many ways to link the corresponding categories in different disciplines. From the double-graph superposition analysis, it can be further seen that the current research presents the situation of interdisciplinary research and the research of a single discipline makes the research of scholars stuck in the bottleneck. With the help of cross-disciplinary technical means, the technical barriers can be broken and the progress of science and technology can be promoted.

### 3.6. Analysis of Hotspots in the Research of Metal–Organic Gels

#### 3.6.1. Keyword Co-Occurrence Network Analysis

**Figure 7:**
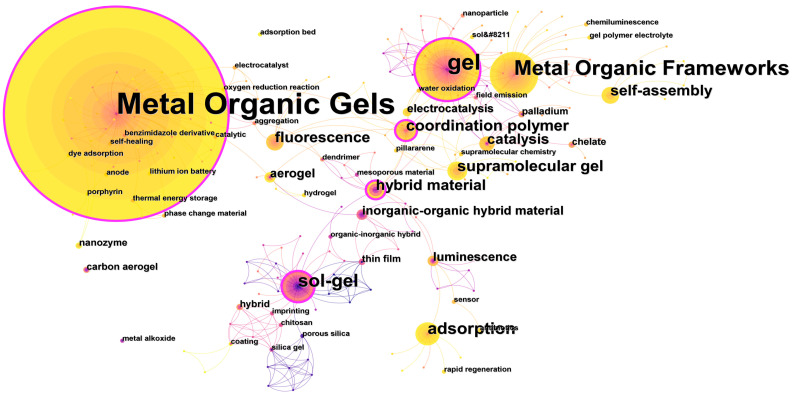
Keyword co-occurrence network diagram in MOGs field.

A research hotspot refers to one or more topics that researchers often pay attention to in a research field, which can show the research direction of scholars in this field. The keywords are the summary and introduction of the main points of the article. If a keyword appears several times in the literature of the research field, the content reflected by the keyword is likely to be a hot topic of research. In Figure 7 and Table 5, the size of the node and the size of the label represent the frequency of keywords, the inner yellow circle represents the research in the recent period of time, the outer purple circle represents the research in the past period, the color of the connecting lines between keywords represents the total number of words, and the thickness represents the co-occurrence frequency.

Through software analysis, a total of 361 keyword nodes and 562 connecting lines composed of them are obtained. It is found that keywords with centrality greater than 0.1 are Metal Organic Gels (0.35), gel (0.28), sol-gel (0.22). coordination polymer (0.31), and hybrid material (0.29); in addition, the frequency of keyword occurrence reflects its research heat in this research field. The keywords in the atlas are counted; the details of the top 12 keywords with the frequency are listed in Table 4, and the results are shown in Figure 7. In addition to the keywords Metal Organic Gels and supramolecular gels, the hot words mainly focus on gel, metal-organic frameworks, sol-gel, adsorption, coordination polymer, hybrid material, self-assembly, fluorescence, and catalysis. By integrating high-frequency keywords, it can be found that MOGs research in recent years mainly focuses on three categories, among which metal organic gels, gel, metal-organic frameworks, supramolecular gel, coordination polymer, and hybrid material are all keywords related to material classification or its derivatives [38,48,49,50,51,52]. Sol-gel and self-assembly are related to the synthesis methods of material. Adsorption, fluorescence, and catalysis are keywords related to material applications [53,54,55,56,57,58].

#### 3.6.2. Keyword Cluster Analysis

**Figure 8:**
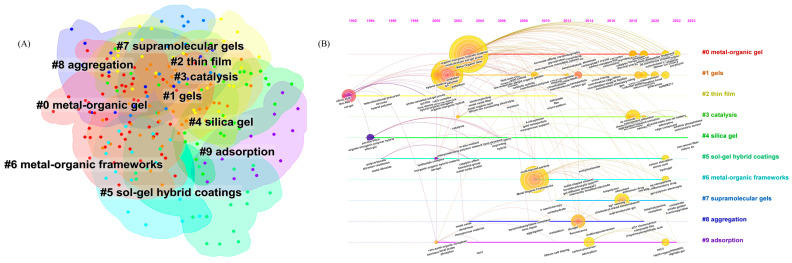
Keyword cluster graph (**A**) and keyword time graph (**B**).

Keywords are the core and essence of a document, which can intuitively show the research theme of a paper. Through the keyword cluster analysis in citespace software, the theme and core ideas of the literature can be roughly understood [59]. The clustering analysis of keywords can better understand the hotspots in the research field. LLR algorithm is used to cluster the keywords of MOGs, and 10 clusters are obtained, as shown in Figure 8A. The information of each cluster is shown in Table 6. It mainly includes #1, gels; #4, silica gel; and #6, metal–organic frameworks. This type of clustering mainly studies the synthesis process of the material itself, mainly involving the selection of metal center and ligand types, proportion, and the characterization of the material itself after synthesis, etc. The practical problems in applications of the second type is based on the materials, including #3, catalysis; # 9, adsorption; # 8, aggregation. This category is mainly combined with the nature of the material itself to deal with practical problems, such as central ion catalytic properties, REDOX properties of lanthanide metal fluorescence properties of ligand functional groups, specific surface area, other properties of materials, and desorption, enrichment of pollutants, or photocatalysis/electrocatalysis to degrade organic macromolecules into inorganic small molecules. In addition, the new trend in this category is fluorescence detection, which uses the properties of lanthanide metals to combine specific functional groups and emit fluorescence of specific wavelength to detect pollutants with extremely sensitive detection limits. The third category mainly includes #5, sol–gel hybrid coatings, which are mainly improved synthesis methods, changing high temperature or toxic and harmful chemical solvents into low energy consumption conditions or green synthesis with no synthetic byproducts, or reducing material synthesis cost, replacing precious rare metals with common-metal salt ions. The fourth category is #2, thin film and membrane separation technology, with the advantages of high separation efficiency, simple operation, no secondary pollution, and low energy consumption, which not only emerged earlier but, in recent years, the majority of scholars have carried out comprehensive in-depth research on the membrane, so some scholars made the metal–organic gel film or load on the film. At present, MOFs are fixed on hydrogels or aerogels, and the direct application of metal–organic gels into membranes has not yet been studied, which needs to be strengthened [60,61,62,63,64,65,66]. Metal–organic gel is a new kind of gel material, and its early achievements are mainly concentrated in the following aspects: 1. Preparation methods of gels: Early studies mainly focused on the preparation methods of metal–organic gels, including solvothermal method, hydrothermal method, solvent volatilization method, etc.; 2. Gel structure and properties: Early studies mainly explored the structure and properties of metal–organic gels, including gel morphology, pore size, surface area, thermal stability, etc.; 3. Application of gels: Early studies also explored the application of metal–organic gels in catalysis, adsorption, separation and other fields, such as catalysts, adsorbents, separation membranes, etc.

The evolution of MOGs keywords evolves with the gradual development of science and technology, which is a dynamic changing process. Figure 8B shows the time diagram of the keywords. Metal-organic gels appeared in 2003 and 2001, respectively, and have a strong correlation with the subsequent studies on catalysis, adsorption and fluorescence. adsorption, luminescence, fluorescence, etc.; all appear after metal-organic gels, indicating a conversion to practical application in the study of the properties of materials. After 2016, the applications are relatively dispersed and diversified, indicating that the field is constantly improving and developing. We summarize the key findings of metal–organic gels as follows:

1. Discovery of metal–organic gels: The earliest metal–organic gels were discovered by Japanese scholar Kato and his team in 1995. Using a mixture of metal ions and organic ligands, they managed to produce a gel-like substance by controlling reaction conditions.

2. Research on gel formation mechanism: With the discovery of metal–organic gels, researchers began to study the mechanism of gel formation. Studies have shown that the interaction between metal ions and organic ligands is a key factor in gel formation. These interactions include hydrogen bonding, van der Waals forces, metal–ligand coordination bonds, etc.

3. Regulation of gel properties: In order to realize the application of metal–organic gels, researchers began to study how to regulate the properties of gel. These properties include gel stability, mechanical properties, optical properties, and so on. The properties of gel can be controlled effectively by adjusting reaction conditions and changing the structure of ligands.

4. Development of applications research: metal–organic gels have a wide range of application prospects in materials science, chemistry, biomedicine, and other fields. Researchers began to study the application of metal–organic gels in these fields, such as sensors, catalysts, drug delivery, etc. These studies provide new ideas and methods for the application and expansion of metal–organic gels.

#### 3.6.3. Keywords Time Zone Map

In order to study the changes of keywords over time, the time graph of keyword network is drawn. From the perspective of time, the evolution of MOGs keywords can be divided into four periods: In the first stage (1992–1999), although the research topics are relatively scattered, they are all related to porous materials and their derivatives. Porous materials are also the source of MOGs, and the research in this stage mainly focuses on the synthesis, characterization, and properties of materials. In the second stage (2000–2015), research topics include metal–organic gels, gels, coordination polymers, metal-organic frameworks, and metal–organic materials with fluorescent adsorption properties for the detection or adsorption of contaminants. This stage of research not only focuses on the synthesis of materials but also includes the modification of materials and load and then applies it to practical problems. The third stage is in recent years (2015 till now): the research topics are distributed in antibiotics, dyes, self-assembly, and supramolecular gel. In this stage, the research on the synthesis mechanism and adsorption mechanism of MOGs begins to be in-depth, involving self-assembly. Hydrogen bond and other keywords, such as pillararene and adsorption bed, mean that adsorption started from simple powders to devices and more mature applications. The appearance of keywords such as lithium battery, anode, and electrocatalysis means that the research field of MOGs has begun to develop into the field of electrochemistry [67,68,69,70,71,72].

#### 3.6.4. Analysis of Burst Keywords

**Figure 9:**
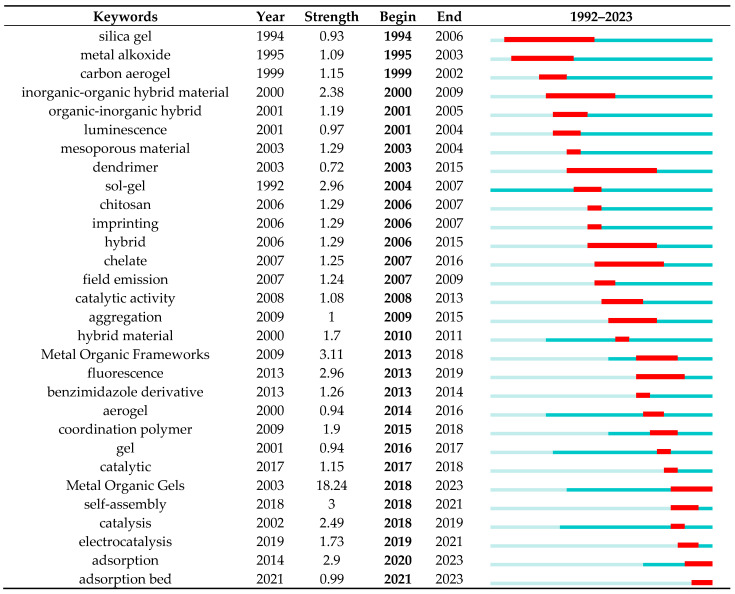
Top 30 keywords with the strongest citation bursts.

Keyword emergence can show the change trend of keywords in this stage over time. Strength refers to the burst intensity, which is realized through the function of burst terms in citespace software. It is used to investigate the terms that appear suddenly and increase rapidly in frequency. Such outburst words often reflect the forefront of research in the field. The blue line represents the time interval, the red line represents the period of the burst keyword, and the end point of the red line represents the beginning and end of each burst interval. “Year” refers to the time of the first occurrence in the search record, and the intensity represents the intensity of keyword occurrence. The greater the intensity of keyword occurrence, the higher the research intensity and research output. In this study, the top 30 keywords with prominent frequency are selected according to Figure 9. According to the graph, the keywords that first appeared in recent years are summarized into periodic research themes and hotspots, and the new possible frontier research problems are explored. According to the retrieved results, the keyword was selected as the node, and the “shortest duration” was set as 1 year. Based on the analysis results of literature emergence words in the Web of Science database, it was found that “metal organic gels” was the most prominent emergence word; the intensity was as high as 18.24, and the breakout period has lasted from 2018 to now. This indicates that current studies mostly focus on the synthesis of materials themselves, followed by metal–organic frameworks, self-assembly, fluorescence, sol–gel, adsorption, and catalysis [73,74], The densities of inorganic–organic hybrid materials were 3.11, 3, 2.96, 2.9, 2.49, and 2.38, respectively. This means that, in addition to the synthesis of materials themselves, the application of materials has also begun to become a research hotspot in the field of metal–organic gels. Studies related to fluorescence first appeared in 2013, catalytic studies first appeared in 2002, and adsorption studies first appeared in 2014. The earliest application of MOGs is similar to that of MOFs. In addition, the field of adsorption and adsorption bed has become a research hotspot in the past 10 years [75,76,77,78]. This field is mainly to shape the synthesized powder material into a material that is not easy to lose and is used to remove related pollutants in practical engineering. In addition, silica gel and dendrimer, which have been studied for the longest time, are related derivatives, but in recent years, research on silica gel and dendrimer decreased [79,80,81]. In addition, catalysis coordination polymer, gel, catalytic, self-assembly, catalysis, electrocatalysis, and other keywords have emerged in the last five years, although catalysis intensity is not high. However, it indicated that the research field began to shift from the application of materials to the molecular level, and gels formed through intermolecular polymerization or self-assembly began to be applied to the field of catalysis. Electrocatalysis is a new research hotspot. The research focus of electrocatalysis can be divided into catalyst research and development and heterogeneous catalysis. By changing the electrode potential on the electrode plate, the structure of the electrode/solution interface double layer, the free energy of the reactant/product, and the adsorption energy of adsorbed ions with certain characteristics will have great changes. At present, most of the high-quality catalysts in electrocatalysis are of nanometer size and most of them are precious metals, which are expensive. Therefore, MOGs formed by common-metal ions instead of precious metals can regulate the surface vacancy (active site) and enhance catalytic performance.

## 4. Summary and Outlook

Through the visual analysis of the literature related to metal–organic gels research in the Web of Science Core collection database, the main conclusions are as follows:

1. The research and development period of MOGs can be divided into the initial stage (1960–1990), the preliminary development stage (1991–2010) and the rapid growth stage (2011–2023). The main countries studied were China, Japan, and France. The main research focuses on Sun Yat-sen University, Southwest University, Chinese Acad Sci, Indian Inst Technol, and Vienna Univ Technol.

2. The top three cited literatures respectively introduced that adding metal and anion to gel factors can further adjust the properties of their materials, including their optical, magnetic, self-assembly, morphological, and rheological behaviors, so as to be applied to different fields. The high surface area, low density, and adjustable porosity characteristics of producing various hierarchical porous Al (III)-MOAs were demonstrated, as well as the design and properties of metal gel types.

3. Based on a review of the history and current situation of bibliometrics research on MOGs, this study proposed the following main research directions in the future:

In the field of adsorption, combined with the time diagram, it can be found that pollutants such as antibiotics and dyes appear in the keywords of the recent five years in the keyword time zone diagram. MOGs themselves are porous materials. Liu et al. [82] synthesized Fe^3+^, Eu^3+^ and TATB into a series of JLUE-MOGs, which have good removal ability of CTC. The powder material is fixed with cellulose, which is used to remove continuous pollutants in the actual wastewater treatment. Wang et al. [83] prepared Zn-MOG by simple fusion method with Zn^2+^ and Xanthine, which showed good removal effect on dye MO. Zheng et al. [84] prepared CAU-3-NH2(gel) with Al^3+^ and NH_2_-BDC for adsorption of toluene in humid air and rapid regeneration at mild temperature. Hong et al. [85] prepared Ni-MOG with Ni^2+^ and BCA, and proved that chelation of metal and ligand played a key role in gel formation. Fang et al. [8] combined Zr^4+^, H2bcpt, and 4, 4-Bpy into HNU-G3. The gel showed significant selectivity and excellent adsorption capacity for Pb (II) (475.88 mg/g), and the adsorbed Pb (II) could be used to synthesize CsPbBr_3_. This paves the way for sustainable use of waste lead (II).

In the field of catalysis, Zhou et al. [86] impregnated photocatalytic active PMA into MOG-Cr. The introduction of PMA promoted the effective separation of electron-hole pairs by capturing and transferring photogenerated electrons. Therefore, the synergistic action of these two components greatly improves the adsorption of some common organic dyes and enhances the oxidative degradation. Jia et al. [86] introduced Ag+ into Mog-Al through in situ growth. Due to the porous and abundant coordination sites in the structural features of MOG, Ag-NP can be easily fixed in MOG with uniform distribution. The resulting Ag-NPs@MOG complex shows significant and persistent activity in the catalytic reduction of p-nitrophenol to aminophenol compounds in aqueous solution. Zhang et al. [87] reported a layered porous metal–metal–porphyrin aerogel (Co-MMPG). The gel retains sufficient conformational flexibility and has binding pockets formed by coplanar arrangements of porphyrin rings. The synergism between the two Co (II) sites in the limited nanospace pocket promotes the binding of different substrates to favorable geometrics, which results in the excellent catalytic performance of Co-MMPG under the background of co-catalysis. Botian et al. [88] used In(III) metal–organic gel as a sacrifice template to prepare indium (III) sulfide mixed gel through in situ polymerization and vulcanization, which can be used to photodegrade RhB and MB, which has potential value in the field of water treatment. Fu et al. [89] prepared a series of new porphyrin-dihydrogen (II) MoGs with porphyrin 1 and Rh2 (OAC) 4, and converted them into corresponding MOA by drying with subcritical CO2 (l).

For the application of MOGs in the field of sensing, Sun et al. [90] designed a new proportional fluorescence probe of Tb-Eu-MOG to realize the analysis and detection of ciprofloxacin in water. Wang et al. [91] synthesized Tb-Ru-MOG at room temperature, which provided a reliable way for the analysis of novel ECL luminators. Xu et al. [92] formed supramolecular gels with BTBM and glacial acetic acid and showed fluorescence emission. After coordination with Pd^2+^, the obtained metal–organic gels could be used to induce CO. Zhao et al. [93] prepared bimetallic organic gel with TATB, Cu(Ⅱ) and Co(Ⅱ) at room temperature, which could catalyze the reaction between H_2_O_2_ and terephthalic acid to produce blue fluorescent products. Qin et al. [94] prepared luminescent TB-based metal–organic gels (MOG (Tb)) by replacing Al ^3+^ in situ with Tb ^3+^ through induction of Al-based MOG to detect antibiotics in the environment.

For the application of MOGs in the electrode field of capacitor batteries, Karan et al. [95] proposed a Zn-MOG that does not require additional electrolytes. Free ions are embedded in the gel, which has self-healing properties and high specific capacitance retention. Sargent et al. [96] synthesized a gelated FeCoW hydroxyl oxide OER electrocatalyst with atomically homogeneous metal distribution. Compared with annealed FeCoW hydroxyl oxide (A- FeCoW) and gel-like FeCoW hydroxyl oxide (G-FeCoW), in alkaline solution, its potential is lower (191 mV). Su et al. [97] prepared spongy porous metal–organic gels with Al^3+^ and H3BTC, which had excellent electrolyte holding capacity and thus retained the characteristics of liquid electrolyte to a large extent. At the same time, MOG electrolyte can penetrate into the photoanode film well, which opens up a new way for efficient preparation of solid-state dye-sensitized solar cells. Dong et Al. [98] added Al^3+^ directly into TBP solution to induce Al^3+^ to form a gel with TBP coordination, which could be used as a gelling agent and active additive to adjust the performance of the electrolyte.

## Figures and Tables

**Table 1:** Information about the top 10 countries in the field of MOGs.

Rank	Countries	Publication Number	Percentage (%)	First Published Year	Centrality
1	China	224	41.71	1998	0.31
2	Japan	54	10.06	2001	0.23
3	India	45	8.38	1994	0.06
4	USA	28	5.21	1993	0.02
5	France	20	3.72	1995	0.18
6	Germany	20	3.72	2000	0.19
7	Spain	17	3.17	1999	0.13
8	South Korea	13	2.42	2000	0.13
9	Russia	12	2.23	1999	0.07
10	Austria	11	2.05	2000	0

**Table 2:** Relevant information of the top 10 authors in the field of MOGs.

Rank	Frequency	Author	Year	Half-Value Period
1	26	Zhang, Jianyong	2009	6.5
2	24	Li, Yuan Fang	2017	1.5
3	16	Huang, Cheng Zhi	2017	1.5
4	10	Su, Cheng-Yong	2009	4.5
5	9	Chen, Liuping	2009	4.5
6	8	Jiang, Zhong Wei	2017	1.5
7	7	Li, Yang	2018	0.5
8	7	He, Li	2017	1.5
9	6	Biradha, Kumar	2012	4.5
10	6	Schubert, U	1994	5.5

**Table 3:** Relevant information of the top 10 institutions in the field of MOGs.

Rank	Frequency	Institution	Year	Half-Value Period
1	26	Sun Yat-sen University	2009	7.5
2	24	Southwest University	2015	3.5
3	16	Chinese Acad Sci	2007	7.5
4	10	Indian Inst Technol	2012	4.5
5	9	Vienna Univ Technol	2000	3.5
6	8	Jilin University	2016	5.5
7	7	Qingdao Univ Sci & Technol	2006	13.5
8	7	Indian Assoc Cultivat Sci	2013	0.5
9	6	Tokyo Inst Technol	2000	9.5
10	6	Kyoto Univ	1998	22.5

**Table 4:** List of co-citation frequencies of MOGs.

Rank	Citations	Author	Year	Journal	Title	Reference
1	72	Piepenbrock	2010	CHEM REV	Metal- and Anion-Binding Supramolecular Gels	[11]
2	60	Li L	2013	NAT COMMUN	A synthetic route to ultralight hierarchically micro/mesoporous Al(III)-carboxylate metal-organic aerogels	[37]
3	54	Zhang JY	2013	COORDIN CHEM REV	Metal-organic gels: From discrete metallogelators to coordination polymers	[38]
4	53	Tam AYY	2013	CHEM SOC REV	Recent advances in metallogels	[39]
5	37	Wei Q	2005	CHEM COMMUN	A metal–organic gel used as a template for a porous organic polymer	[40]
6	33	Lohe MR	2009	CHEM COMMUN	Metal–organic framework (MOF) aerogels with high micro- and macroporosity	[41]
7	32	Sutar P	2016	CHEM COMMUN	Coordination polymer gels: soft metal–organic supramolecular materials and versatile applications	[42]
8	32	Samai S	2012	CHEM MATER	Chemical and Mechano Responsive Metal–Organic Gels of Bis(benzimidazole)-Based Ligands with Cd(II) and Cu(II) Halide Salts: Self Sustainability and Gas and Dye Sorptions	[43]
9	32	Sangeetha	2005	CHEM SOC REV	Supramolecular gels: Functions and uses	[44]
10	30	Xiang SL	2012	J MATER CHEM	Porous organic–inorganic hybrid aerogels based on Cr^3+^/Fe^3+^ and rigid bridging carboxylates	[45]

**Table 5:** Top 12 keywords in terms of frequency in MOGs.

Rank	Frequency	Keywords	Centrality
1	101	Metal Organic Gels	0.35
2	29	gel	0.28
3	22	Metal Organic Frameworks	0.09
4	14	sol-gel	0.22
5	11	adsorption	0.03
6	9	supramolecular gel	0.03
7	9	coordination polymer	0.31
8	8	hybrid material	0.29
9	8	self-assembly	0.03
10	8	fluorescence	0.03
11	7	catalysis	0.03
12	5	inorganic-organic hybrid material	0.03

**Table 6:** Clustering information of MOGs.

ID	Number of Nodes	Silhouette	Average Years	Main Content
0	52	0.973	2017	metal–organic gel (17.65, 0.0001); metallogel (10.32, 0.005); metal–organic gels (7.84, 0.01); thermal energy storage (6.86, 0.01); catalytic (6.86, 0.01)
1	42	0.911	2012	gels (37.64, 0.0001); chelates (14.12, 0.001); pillararenes (9.38, 0.005); coordination polymers (9.38, 0.005); nanoparticles (9.38, 0.005)
2	30	0.995	1999	thin film (19.69, 0.0001); sol–gel (17.06, 0.0001); pzt films (6.49, 0.05); moisture (6.49, 0.05); surface (6.49, 0.05)
3	18	0.924	2017	catalysis (9.46, 0.005); self-assembly (9.46, 0.005); bifunctional oxygen electrocatalyst (6.56, 0.05); 4-nitrophenol (6.56, 0.05); organophosphorus pesticides (6.56, 0.05)
4	18	0.994	2002	silica gel (11.75, 0.001); transition metal salts (7.69, 0.01); polyoxazoline (7.69, 0.01); pyrolysis (7.69, 0.01); organic–inorganic (7.69, 0.01)
5	18	0.993	2004	sol–gel hybrid coatings (7.56, 0.01); functional groups (7.56, 0.01); metal alkoxide (7.56, 0.01); organoalkoxysilanes (7.56, 0.01); abrasion resistance (7.56, 0.01)
6	17	0.967	2015	metal–organic frameworks (14.61, 0.001); metal–organic framework (12.61, 0.001); heavy metal ion (6.27, 0.05); drug loading (6.27, 0.05); assembly (6.27, 0.05)
7	14	0.950	2017	supramolecular gels (10.75, 0.005); glucosamine (7.21, 0.01); carbohydrate (7.21, 0.01); Ag^+^ sensing (7.21, 0.01); ionic conductivity (7.21, 0.01)
8	13	0.941	2011	aggregation (15.25, 0.0001); sol–gel processes (11.48, 0.001); fluorescence (9.79, 0.005); ionic liquids (7.56, 0.01); benzimidazolylidene complexes (7.56, 0.01)
9	13	0.985	2010	adsorption (13.9, 0.001); phosphor (13.9, 0.001); luminescence (13.9, 0.001); in situ synthesis of metal–organic frameworks (6.9, 0.01); alginate gel (6.9, 0.01)

## Data Availability

The data presented in this study are available on request from the corresponding author.
